# Polytrauma in geriatric patients: incidence and mortality

**DOI:** 10.1186/1471-2318-11-S1-A13

**Published:** 2011-08-24

**Authors:** F Famà, L M Murabito, A Beccaria, F Cucinotta, A Caruso, C D Foti, G Versace, N La Torre, C Estollere, P Placanica, M A Gioffrè Florio

**Affiliations:** 1 Unità Operativa Complessa di Medicina e Chirurgia d’Accettazione e d’Urgenza con Osservazione Breve - Azienda Ospedaliera Universitaria “G.Martino” di Messina, Italy

## Background

The increase of traumatic events involving geriatric patients is due to longer life expectancy and a progressive improvement of quality of life. More and more elderly subjects carry out various activities, however because of physical frailty, activity exposes them to the risk of injuries and accidents. They are more susceptible to traumatic injuries of road traffic accidents. Our objective was to analyze how increased age and the presence of several related diseases have contributed to an increase in the incidence of multiple trauma in the last 3 years.

## Materials and methods

**Figure 1:**
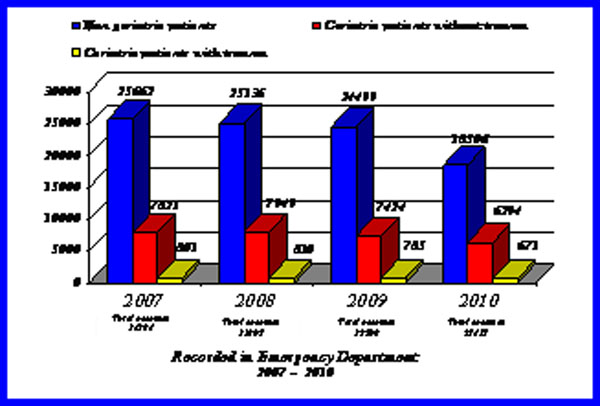
Injuried patients recorded in our Emergency Department from 2007 to 2010

In the period January 2007 - October 2010, in our UOC MCAU with OB, adequately equipped for major trauma with a Trauma area and RED area with two Shock Rooms, 126304 total referrals were recorded. The percentage of geriatric patients (≥65 years) was 25.73% (N=32501), amongst these 3067 (2.42% of total referrals) had medium-severe injuries from domestic or road traffic accidents (Fig.1).

## Results

**Figure 2:**
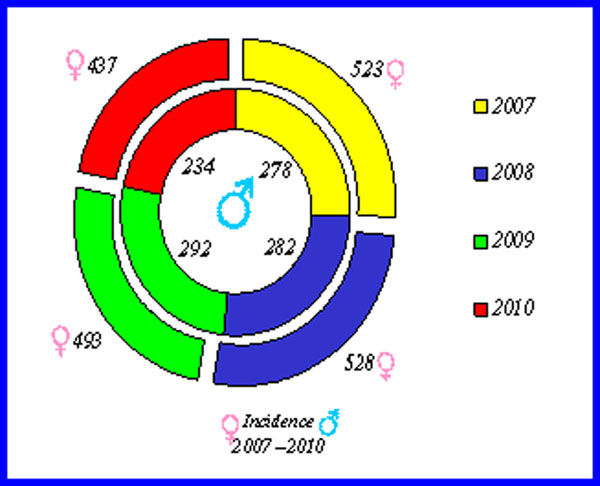


**Figure 3:**
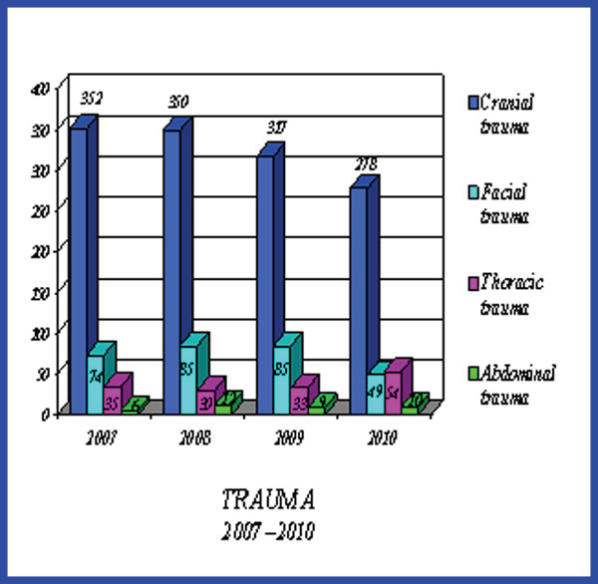


We observed that the incidence of geriatric trauma has remained constant every year, with a majority of females over males (F/1981-M/1086) (Fig.2) and that the age group most affected is between 75 and 84 years. The 3067 patients we observed: head trauma was present in 1297 patients (42.3%), head and/or facial trauma 293 (9.6%), followed by thoracic trauma 242 (7.9%) and/or abdominal trauma 37 (1.2 %), often associated with single or multiple fractures (Fig.3).

The most recurrent fracture was the fracture of the femur 513 (16.72%), especially in women (possibly secondary to osteoporosis).

Also numerous were fractures of the upper limbs (N=474) and rib fractures (N=333). In 40% of cases (1227) the trauma was related to a syncope event. 1981 patients required hospitalization, predominantly in Orthopaedics and Traumatology (N=582), in neurological wards (N=170), in Thoracic Surgery (N=107) and 235 in other departments. In the Emergency Department (UOC MCAU with OB), no death for geriatric trauma was observed.

## Conclusions

The most frequent traumatic event is represented by falls, often associated with syncopal events, very frequent syndrome in geriatrics. In road traffic trauma, the geriatric patient is more often a pedestrian, therefore, the risk of back injuries and fractures is significantly higher, and the risk of permanent disability or death is high.
